# 

**Published:** 1868-02

**Authors:** 


### CONTENTS.

ORIGINAL COMMUNICATIONS.

Microscopy of the teeth .......... 51
Address to the Graduating Class of the Ohio Dental College .......... 55
Address of the Retiring President, Dr. W. H. Goddard .......... 60
Indiscriminate Extraction of the Six Year Molars is a Sin, and should be Condemned .......... 67
On the use of Oxy-Chloride of Zinc over Exposed Pulps .......... 69
The Effects of Light upon Chloroform—Important suggestion to those Using the Article .......... 71

PROCEEDINGS OF SOCIETIES.

Minutes of the Second Annual Meeting of the Ohio State Dental Society .......... 73
East Tennessee Dental Association .......... 76
Proceedings of the Central States Dental Society .......... 77
Proceedings of the Ohio State Dental Association .......... 81
Notice .......... 85
Western New York Dental Association .......... 85

SELECTIONS.

A Just Tribute .......... 87

Practical Dentistry—An Important Movement .......... 88

EDITORIAL.

Dental Bill .......... 91
The Rubber Contest .......... 91
Obituary .......... 91

